# Editorial: Genetic and cellular heterogeneity in tumors

**DOI:** 10.3389/fcell.2024.1519539

**Published:** 2024-12-09

**Authors:** Zishan Wang, Li Ma, Juan Xu, Chunjie Jiang

**Affiliations:** ^1^ Department of Genetics and Genomic Sciences, Department of Artificial Intelligence and Human Health, Center for Transformative Disease Modeling, Tisch Cancer Institute, Icahn Genomics Institute, Icahn School of Medicine at Mount Sinai, New York, NY, United States; ^2^ Department of Microbiology, Immunology and Cell Biology, West Virginia University, Morgantown, WV, United States; ^3^ College of Bioinformatics Science and Technology, Harbin Medical University, Harbin, Heilongjiang, China; ^4^ Department of Genomic Medicine, The University of Texas MD Anderson Cancer Center, Houston, TX, United States

**Keywords:** tumor, heterogeneity, genetic, tumor microenvironment, invasiveness, metastasis, prognosis

Tumorigenesis is a heterogenous process, promoted not only by genetic mutations within cells, but also by the outer tissue space feeding the mutated cells - tumor microenvironment (TME). Different genetic mutations can drive tumorigenesis by perturbing distinct pathways that enable tumor occurrence (Ostroverkhova et al., 2023). TME consists of tumor cells interacting with diverse non-tumorigenic components, including immune cells, stromal cells, and other cell types, as well as extracellular matrix (Anderson and Simon, 2020). These components form a supportive niche that facilitate tumor cell survival, proliferation and metastasis (de Visser and Joyce, 2023; Li et al., 2024). The intermix of genetic diversity and TME variability leads substantial challenges for tumor treatment, as tumor cells can evade therapeutic interventions by exploiting alternative signaling pathways or adopting protective states within the TME (Vinay et al., 2015; Sun, 2016; Baghban et al., 2020; Yip and Papa, 2021). Thus, understanding tumor heterogeneity is essential for developing effective, personalized tumor treatment.

To address these challenges, numerous technologies have been developed, such as microarray, next-generation sequencing, single-cell sequencing, spatial omics, mass spectrometry, 3D cell culture systems, and advanced imaging technologies. Large-scale national projects like The Cancer Genome Atlas Program (TCGA) (Cancer Genome Atlas Research Network et al., 2013), Clinical Proteomic Tumor Analysis Consortium (CPTAC) (Ellis et al., 2013), and Human Tumor Atlas Network (HTAN) (Rozenblatt-Rosen et al., 2020) have leveraged these technologies to characterize the molecular and cellular landscape of various tumors. As the cost of these technologies decrease, more researchers conduct in-depth studies, accumulating unprecedented datasets that enhance our understanding of tumor heterogeneity and facilitate the development of personalized treatments.

The Research Topic entitled “Genetic and Cellular Heterogeneity in Tumors” focuses on characterizing the genetic mechanism or TME variations that contribute to tumor heterogeneity complicating treatments, and new techniques/methods of exploring such. Here, we gathered four articles of breast cancer or acute myeloid leukemia (AML) examining genetic alterations or cell communications within TME, and their implications with tumor invasion, metastasis, and prognosis.

Breast cancer is one of the most prevalent tumors, significantly contributing morbidity or mortality and becoming an urgent health concern (Sung et al., 2021). Triple negative breast cancer (TNBC), occupying 10%–20% of invasive breast cancer cases, is the subtype with the worst prognosis caused by the absence of targeted therapeutic options (Kumar and Aggarwal, 2016). Bone morphogenetic protein (BMP) signaling has been implicated in the progression and metastasis of breast cancer, wherein high expression BMP8A revealed to be correlated with poor survival (Katsuta et al., 2019). Sui et al. investigated the role of BMP8A in the progression of TNBC, emphasizing its involvement in bone metastasis. An elevated expression of BMP8A was observed in TNBC cohort from TCGA, corroborated by the immunohistochemical staining experiment, and expression of BMP8A was associated with patient’s reduced survival. *In vitro* cellular function tests conducted in TNBC cell lines, MDA-MB-231 and BT549, demonstrated that BMP8A overexpression was accompanied with cell invasion and migration. Additionally, BMP8A expression was positively correlated with markers from Epithelial-Mesenchymal Transition (EMT), a key processes facilitating tumor cell motility (Dongre and Weinberg, 2019), and Matrix Metalloproteinases (MMPs), which are thought to affect cell behaviors including tumor spread (Stamenkovic, 2003), suggesting that BMP8A may enhance invasiveness of TNBC cells by regulating EMT and MMPs. The study observed a high correlation between BMP8A expression and key biomarkers associated with bone metastases, especially the osteolytic factors of RANKL, a key component in the RANK-RANKL-OPG system that are associated with bone metabolism and mammary epithelial cell development. Taken together, Sui et al. revealed relevance of BMP8A overexpression with tumor invasiveness and bone metastasis, indicating its therapeutic potential in TNBC.

Metastatic breast cancer accounts for more than 10% of patients, which is the leading cause of death in this population (Scully et al., 2012; Esposito et al., 2021). Similar to TNBC, the reason of such high death is partly attributed to lacking targetable genetic vulnerability of metastasis. While it is believed that only a subset of genetically predisposed tumor cells metastasize, deeper insights into genetic heterogeneity benefits personalized treatment of metastatic breast cancer (Basho and Chase, 2023). Lake et al. achieve this at certain degree by combing an organoid-based breast cancer metastatic mice model with digital droplet polymerase chain reaction (ddPCR) to investigate genes whose copy number amplifications (CNA) identified to be associated with breast cancer metastasis. Their methods focused on CNA invasiveness potential of FGFR1, the most clinically mature targets identified in their analysis. They found that invasive organoids display statistically significant copy number amplification, demonstrating that higher CNA of FGFR1 correlates with organoid invasion. The organoid-ddPCR model in this study provides a robust method to capture tumor heterogeneity and evaluate therapeutic response, with significant implications on clinical practice and cancer biology.

In addition to genetic heterogeneity, variations in TME also influence breast cancer progression and treatment outcomes (Desai et al., 2024). Characterizing the interactions between distinct cells in TME may reveal the critical breast cancer vulnerabilities and provide novel diagnostic and therapeutic perspectives (Li et al., 2021). Han et al. reviewed the interplay between myeloid-derived suppressor cells (MDSCs) and platelets, as well as their effects on the breast cancer TME of immune, metabolism, and angiogenesis. MDSCs, known for one of the most effective immunosuppressive cell types, play critical roles in tumor progression and therapeutic strategy (Li et al., 2023). Tumor-associated platelets (TAPs) contribute to immune evasion and tumor spread (Chen et al., 2023). They also summarize existing preclinical and clinical studies, traditional Chinese medicine therapeutic approaches, and emerging technologies related to targeting and preventing the interaction of MDSCs with TAPs in TME, and discussed the potential mechanisms and perspective for future. Further investigation into the complexity and heterogeneity of MDSCs and side effects of antiplatelet agent is still required for effective strategy development.

Given the large impact of distinct cell types in TME onto tumor treatment, Han et al. proposed a score system called leukemic stem cells score (LSCA) to predict the prognosis of acute myeloid leukemia (AML) patient in terms of the expression-deconvoluted abundance of cells in TME. AML is the most common type of acute leukemia in adults and characterized by the immature differentiation of myeloid cells (De Kouchkovsky and Abdul-Hay, 2016). Leukemic stem cells (LSCs) are believed to be a major contributor to leukemia progression and drug resistance (Vetrie et al., 2020; Zhai and Jiang, 2022), but the influence of LSCs within TME on patient survival remains inadequately investigated. Currently single cell analysis in large scale hematologic malignancy is limited, and expression-based model to predict prognosis is prevalent. Thus, expression-based cellular deconvolution may be informative in forecasting AML prognosis. Han et al. applied an expression-based method, CIBERSORT (Newman et al., 2015), to hundreds of AML samples and inferred 9 cell-type fractions, subjected to further feature selection. Five cell types exhibiting significance of estimate coefficients, including granulocyte-monocyte progenitors (GMPs), common myeloid progenitors (CMPs), CD45RA + cells (RApos), megakaryocyte-erythrocyte progenitors (MEPs), and multipotent progenitors (MPPs), were selected to calculate the LSC activity (LSCA) score of predicting prognosis. LSCA successfully stratifies patients with distinct survival across cohorts, where patients with lower LSCA scores showed favorable clinical outcomes. The area under the curves (AUCs) analysis indicated the performance of LSCA score system was comparable to existing prognostic models, LSC17 (Ng et al., 2016), APS (Docking et al., 2021), and CTC score (Dai et al., 2021), suggesting its utility as a prognostic tool for tumor.

This Research Topic provides valuable insights into genetic-driven and TME-driven tumor heterogeneity that influence progression and therapeutic strategy in tumor. Studies on BMP8A’s role in TNBC invasiveness, FGFR1 genetic amplification in metastatic breast cancer, MDSC-platelet interactions in breast cancer TME, and the development of the TME-cell-abundance-based LSCA score for AML prognosis all underscore the importance of tumor heterogeneity in tumor research. These discoveries result from advanced development of different technologies. The rapidly evolving technologies will gain deeper insights into genetic/TME heterogeneity at a finer resolution and pave a smooth way for the next-generation of personalized effective treatment in tumor.
